# Separate and combined effects of advanced age and obesity on mammary adipose inflammation, immunosuppression and tumor progression in mouse models of triple negative breast cancer

**DOI:** 10.3389/fonc.2022.1031174

**Published:** 2023-01-04

**Authors:** Laura A. Smith, Dalton M. Craven, Magdalena A. Rainey, Alyssa J. Cozzo, Meredith S. Carson, Elaine M. Glenny, Nishita Sheth, Shannon B. McDonell, Erika T. Rezeli, Stephanie A. Montgomery, Laura W. Bowers, Michael F. Coleman, Stephen D. Hursting

**Affiliations:** ^1^ Department of Nutrition, University of North Carolina at Chapel Hill, Chapel Hill, NC, United States; ^2^ Department of Pathology and Laboratory Medicine, University of North Carolina at Chapel Hill, Chapel Hill, NC, United States; ^3^ Lineberger Comprehensive Cancer Center, University of North Carolina at Chapel Hill, Chapel Hill, NC, United States; ^4^ Nutrition Research Institute, University of North Carolina at Chapel Hill, Chapel Hill, NC, United States

**Keywords:** breast cancer, obesity, advanced age, tumor immunosuppression, inflammation

## Abstract

**Introduction:**

Advanced age and obesity are independent risk and progression factors for triple negative breast cancer (TNBC), which presents significant public health concerns for the aging population and its increasing burden of obesity. Due to parallels between advanced age- and obesityrelated biology, particularly adipose inflammation, we hypothesized that advanced age and obesity each accelerate mammary tumor growth through convergent, and likely interactive, mechanisms.

**Methods:**

To test this hypothesis, we orthotopically transplanted murine syngeneic TNBC cells into the mammary glands of young normoweight control (7 months), young diet-induced obese (DIO), aged normoweight control (17 months), and aged DIO female C57BL/6J mice.

**Results:**

Here we report accelerated tumor growth in aged control and young DIO mice, compared with young controls. Transcriptional analyses revealed, with a few exceptions, overlapping patterns of mammary tumor inflammation and tumor immunosuppression in aged control mice and young DIO mice, relative to young controls. Moreover, aged control and young DIO tumors, compared with young controls, had reduced abundance ofcytotoxic CD8 T cells. Finally, DIO in advanced age exacerbated mammary tumor growth, inflammation and tumor immunosuppression.

**Discussion:**

These findings demonstrate commonalities in the mechanisms driving TNBC in aged and obese mice, relative to young normoweight controls. Moreover, we found that advanced age and DIO interact to accelerate mammary tumor progression. Given the US population is getting older and more obese, age- and obesity-related biological differences will need to be considered when developing mechanism-based strategies for preventing or controlling breast cancer.

## 1 Background

In women, breast cancer (BC) is the most commonly diagnosed cancer and has the second highest mortality rate of all cancers (1). Two major risk factors for BC are obesity and advanced age. This confluence of factors presents a critical public health concern due to global increases in both obesity rates and population aging (2, 3). In the United States, 42.4% of adults were classified as obese in 2017–2018 (4), and in North America, the proportion of individuals 65 and older is projected to rise from 18% in 2019 to 26% by 2050 (3).

Obesity not only increases risk of BC development, predominantly in postmenopausal women, but also confers worse disease outcomes, including greater mortality and shorter time-to-disease recurrence, in both premenopausal and postmenopausal patients with BC (5, 6). Obesity-related increases in BC incidence and progression are recapitulated in preclinical models of BC, which has facilitated investigation into underlying mechanisms (7, 8). Obesity enhances BC progression, in part, through propagation of low-grade, chronic inflammation within the mammary adipose microenvironment (9). Expansion of adipose tissue in response to overnutrition increases adipocyte dysfunction and death as well as release of cytokines and cellular contents into the surrounding tissue (9). Multiple inflammation-related pathways are thus triggered, including: i) NOD-like receptor signals in response to release of damage-associated proteins; ii) Toll-like receptors in response to free fatty acids; and iii) NF-κB signaling in response to cytokines and chemokines that facilitate the recruitment and activation of proinflammatory immune cells (9, 10). Constitutive stimulation of the immune system during chronic, low-grade inflammation promotes an adipose microenvironment abundant in proinflammatory mediators and immunosuppressive cell types conducive for rapid tumor growth (10). Proinflammatory cytokines directly stimulate tumor cell proliferation and indirectly modify vasculature and extracellular matrix within the mammary microenvironment to enhance tumor growth and metastatic potential (9). Immune suppressor cells, including T regulatory cells, suppress key immune cells that participate in antitumor immunity including antigen-presenting dendritic cells and cytotoxic CD8 T cells (11). Moreover, obesity accelerates mammary tumor progression in association with upregulation of immune checkpoint pathways in effector T cells and increased frequency of exhausted T cell phenotypes (12, 13).

As with obesity, advanced age is also associated with propagation of systemic and adipose-specific low-grade, chronic inflammation and immune dysfunction (14). However, the impact of advanced age on BC progression and the tumor microenvironment has been less studied than the obesity–BC link. Epidemiological data supports advanced age-related increases in BC incidence and mortality as approximately 45% of BC diagnoses and 60% of BC-related deaths occur in women aged 65 years or older (15). Two clinical studies demonstrate that among patients with the same BC subtype, specifically triple negative (TNBC), advanced age at diagnosis increased BC-specific mortality compared with younger patients (16, 17). Thus, when tumor intrinsic features are similar, tumor extrinsic features within the aged host contribute importantly to BC progression.

In light of the biological hallmarks shared between obesity and aging, including chronic inflammation and immune dysfunction (14), we posited that advanced age and obesity each promote BC through similar tumor-extrinsic mechanisms, and may interact. To test our hypothesis we utlized murine models of diet-induced obesity (DIO), ageing, and TNBC.

## 2 Methods

### 2.1 Cell lines

E0771 mammary carcinoma cells, purchased from ATCC (#CRL-3461; Gaithersburg, MD), and metM-Wnt^lung^ mammary carcinoma cells, developed in the Hursting laboratory, were labeled with lentiviral vector containing eGFP and NanoLuc (gifted by Dr. Antonio Amelio) (18), and were maintained in RPMI1640 media (GIBCO Life Technologies, Waltham, MA) supplemented with 10% FBS, 10 mmol/L HEPES buffer, 2 mmol/L L-glutamine, and 1% penicillin/streptomycin. Cells were confirmed *via* the Universal Mycoplasma Detection Kit (ATCC, #30-1012K) to be negative for mycoplasma. Bot hcell lines were also confirmed *via* Affymetrix Mouse Clariom S assay-HT (Thermo-Fischer, Waltham, MA) to be estrogen receptor-, progesterone receptor- and Her2-negative.

### 2.2 Animal studies

All animal protocols were approved by the University of North Carolina at Chapel Hill Institutional Animal Care and Use Committee and carried out in compliance with all guidelines and regulations.

#### 2.2.1 Advanced age and DIO effects on tumor progression

Using 2 distinct murine models of TNBC (study A: metM-Wnt^lung^ cell orthotopic transplant model (8); study B: E0771 cell orthotopic transplant model), we evaluated the impact on tumor progression of advanced age and DIO alone (study A) and alone or in combination (study B, 2x2 factorial design). To generate aged cohorts of normoweight control and obese mice, 6–8-week-old female C57BL/6J mice were purchased from the Jackson Laboratory (Bar Harbor, ME; #000664), and on arrival placed on either a low-fat, control diet (n=44; 10% kcal from fat; Research Diets Inc., New Brunswick, NJ; #D12450J) or high-fat, DIO diet (n=20; 60% kcal from fat; #D12492), respectively. When aged cohorts were 12 months old, young cohorts of normoweight control and obese mice were generated by purchasing additional 6–8-week-old female C57BL/6J mice (The Jackson Laboratory) and administering them control or DIO diets (n=30/group). Four months later, blood was collected from all mice *via* submandibular bleed, and serum was processed and stored for subsequent marker analyses. One month after blood collection, mice were randomized for use in Study A or B as described below. Prior to randomization, 2 young control and 2 aged DIO mice had died from unknown causes, and 3 aged DIO mice had been euthanized due to severe head tilt or dermatitis. The aged (i.e., 17 month-old) control mice, young (i.e., 7 month-old) control mice, and young DIO mice were randomized, by group, evenly to Study A or B. Aged DIO mice were all assigned by design to Study B.

In Study A, mice in each of 3 groups (aged control, young control, and young DIO) received orthotopic injection of 1.5 x 10 (4) metM-Wnt^lung^ cells/50μL PBS into their fourth mammary fat pad (MFP). Mice were palpated twice weekly for 3 weeks, and tumor sizes were measured with electronic calipers until the majority of one of the experimental groups reached maximum allowable size, defined as a diameter of 1.5 cm measured in any direction. Tumor volume (V) was calculated using tumor length (L, the greatest diameter), tumor width (W, the diameter perpendicular to L), and the equation V=L x W (2) x 0.5. Censored during the tumor-monitoring phase were 1 young control, 4 young DIO, and 6 aged control mice due to severe tumor ulceration;1 aged control mouse for severe dermatitis; 1 aged control mouse for poor health; and 1 young control mouse for death by unknown cause. At the end of study, all living mice were euthanized, tissues collected (including tumors, MFPs, and lung), and tumors weighed. Half of each tissue was flash frozen in liquid nitrogen for biochemical or molecular analysis, and half was formalin fixed and paraffin embedded (FFPE) for histological analysis.

In Study B, mice in each of 4 groups (aged control, young control, young DIO, and aged DIO) received orthotopic injection of 1.2 x 10 (4) E0771 cells/50 μL PBS into their fourth MFP. All monitoring and other procedures were identical to those done in Study A. Censored during the tumor-monitoring phase were 1 young control and 1 aged DIO mice due to severe tumor ulceration; 1 young control, 1 young DIO, and 3 aged DIO mice for severe dermatitis; 1 aged DIO for poor health, and 1 aged DIO mouse for death by unknown cause.

#### 2.2.2 CD8 depletion experiment

Female C57BL/6 mice (2 months old, n=54; Jackson Laboratory, #000664) were purchased, placed on the low-fat control diet, and allowed to acclimate for one week. Mice received either intraperitoneal injection with PBS (n=6), 200 μg anti-mouse CD8α-depleting antibody (n=24; BioXCell, Lebanon, NH; clone 2.43, #BE0061), or rat IgG2B isotype (n=24; BioXCell; LTF-2, #BE0090) 2 days prior to tumor cell injection, the day of tumor cell injection, and every 4 days following tumor injection for up to 8 treatments. All mice were injected with 1.5 x 10 (4) metM-Wnt^lung^ cells/50 μL PBS into the fourth MFP. Tumor growth was monitored twice weekly using electronic calipers, with tumor volume calculated as described above. To determine efficacy of the CD8α antibody and duration of CD8+ T-cell depletion, 6 randomly chosen mice from the groups receiving anti-mouse CD8α antibody or IgG2B isotype were euthanized at 13, 17, 21, and 25 days following tumor inoculation, and tumors and spleens collected. The mice receiving PBS injections were all killed at 25 days. At 2 timepoints, 17 and 25 days following tumor inoculation, tumors from the 6 randomly chosen mice per group were dissociated into a single cell suspension using Miltenyi Biotec’s (Bergisch Gladbach, Germany) Tumor Dissociation kit (#130-096-730) and gentleMACs Dissociator (#130-093-235), according to manufacturer’s protocol, and splenocytes were isolated as described previously (19). Single cell suspensions were treated with TruStain FcX (BioLegend, San Diego, CA; #101320) to bind Fc receptors and block nonspecific binding to immunoglobulins and then stained with Zombie UV fixable viability dye (BioLegend; #423108) and antibodies against CD45 (BioLegend; #103106), CD3 (BioLegend; #100306), and CD8 (BioLegend; #100712). Fluorescence intensity was measured using Becton Dickinson LSRII through the UNC Flow Cytometry Core Facility.

### 2.3 Body composition measures

Body composition was assessed *via* quantitative magnetic resonance imaging (Echo Medical Systems, Houston, TX) prior to tumor inoculation and at endpoint. Imaging resulted in measurements of lean mass, fat mass and total water mass. Body fat mass and lean mass percentages were calculated by dividing fat mass and lean mass, respectively, by total body weight.

### 2.4 Tumor immunohistochemistry

FFPE tumors (8 randomly selected mice/group) were sectioned and mounted on slides. Two tumor sections 100 μm apart were stained *via* IHC for antibodies against Ki67 (Vector Laboratories, Burlingame, CA; #VP-RM04), CC3 (Cell Signaling, Danvers, MA; #9661), CD3 (Abcam, Cambridge, MA; #ab16669), and CD8 (Cell Signaling, #98941S). Staining was analyzed using QuPath software (20).

### 2.5 Metastasis analysis

FFPE lungs (8 randomly selected mice/group from study B) were sectioned and stained with hematoxylin and eosin or anti-green fluorescent protein (GFP; Aves Labs, Tigard, OR; #GFP-1020). Stained lung sections were imaged using Aperio ImageScope (Leica Biosystems, Wetzlar, Germany) and the number of mice that developed at least 1 metastatic lesion was quantified by a veterinary pathologist (coauthor Stephanie Montgomery, DVM, PhD, Director, UNC Animal Pathology Core).

### 2.6 RNA profiling and gene set enrichment analysis

Total RNA was extracted from flash-frozen tumor-adjacent MFP (TA-MFP) and tumor samples using TRIzol Reagent (Invitrogen, Carlsbad, CA; #15596026) and RNeasy Mini Kit (Qiagen, Hilden, Germany; #74104) according to manufacturer’s instructions. Adjacent mammary and matched tumor samples (n=6/group) were selected from mice with tumor masses closest to the median of their experimental group. RNA quality was assessed using an Agilent 2100 Bioanalyzer. Gene expression profiling was performed by the Functional Genomics Core at the University of North Carolina. In brief, GeneChip™ WT PLUS Reagent Kit (Applied Biosystems, Carlsbad, CA; #902414) was used to synthesize fragmented and labeled sense-strand cDNA from total RNA, according to the manufacturer’s protocol. Fragmented and labeled cDNA was used to prepare a hybridization cocktail with the Affymetrix GeneTitan Hybridization Wash and Stain Kit for WT Arrays (#901622). Hybridization, washing, staining and scanning of the Affymetrix Clariom™ S Assay HT (#902972) was carried out using the Affymetrix GeneTitan Instrument (Applied Biosystems). Transcriptome Analysis Console Software v 4.0 (Applied Biosystems) was used for basic data analysis and quality control.

Bulk gene expression data was analyzed using GSEA in combination with Hallmark and C7 Immunologic gene sets (21–23). In Study A, two phenotypic comparisons were performed: aged control vs young control, and young DIO vs young control. In Study B, one phenotypic comparisons was performed: aged control vs aged DIO. Statistically significant gene set enrichment was defined by false discovery rate (FDR) q-value < 0.05. Gene sets commonly enriched or underrepresented across comparisons were identified and hypergeometric analysis performed to test significance of the overlap. Leading edge analysis (LEA) was performed on gene sets commonly enriched or underrepresented across comparisons (21, 22). The probability of overlapping gene set enrichment and leading edges was computed *via* hypergeometric analysis. Spearman correlation analyzed differential contribution of each leading edge gene to enrichment of gene sets across the two pair-wise comparisons.

### 2.7 Statistical analysis

Animal study data are presented as mean ± SD. Statistical analyses were performed using GraphPad Prism software (GraphPad Software Inc., San Diego, CA) and statistically significant differences defined by p <0.05. Differences across two groups were analyzed by t-test. Differences across three or more experimental groups were analyzed using one-way ANOVA, followed by Tukey *post hoc* test, if comparing all means to young control, or Dunnett *post hoc*, if comparing each mean to every other mean.

## 3 Results

### 3.1 Advanced age and DIO each promote primary tumor growth in a metM-Wnt^lung^ cell-driven model of TNBC

**Figure 1:**
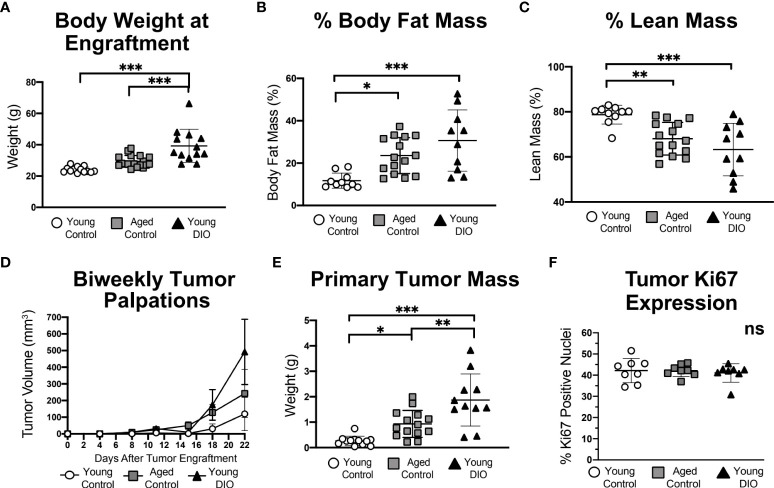
Advanced age and DIO increase mammary tumor growth in a metM-Wnt^lung^ cell transplant model of TNBC. **(A)** Body weight, **(B)** percent body fat mass and **(C)** percent lean mass measured prior to tumor engraftment. **(D)** *In vivo* tumor volume. **(E)** *Ex vivo* primary tumor mass. **(F)** Percentage of cells stained positive for Ki67 *via* immunohistochemistry, analyzed using QuPath. Data presented as mean ± SD. Differences across all groups analyzed using one-way ANOVA and Tukey *post hoc* test. Asterisks indicate differences in significance: *p < 0.05, **p < 0.01, ***p < 0.001. ns, non-significant.

To test whether advanced age and DIO exert similar enhancing effects on progression of Wnt-driven TNBC, three experimental groups were evaluated following engraftment with metM-Wnt^lung^ cells (Study A): 1) young mice fed a low-fat, control diet (young control), 2) aged mice fed a low-fat control diet (aged control), and 3) young mice fed a high-fat, DIO diet (young DIO). The DIO diet, compared with control diet, resulted in increased body weight and percentage body fat mass, and decreased percentage lean mass, in young mice (**Figure 1A-C**). Advanced age, relative to young age, did not significantly affect body weight (**Figure 1A**), yet increased percentage body fat (**Figure 1B**) and reduced percentage lean mass (**Figure 1C**), in control-fed mice. The effects of advanced age on body composition were less pronounced compared to the effects of DIO.

Mammary tumors grew in each group (**Figure 1D**), becoming significantly more massive in aged control mice and young DIO mice than young control mice by study end (**Figure 1E**). Average final tumor weights ranked in the following order, by group: young control < aged control < young DIO. Despite group-dependent differences in tumor sizes, no between-group differences were detected in tumoral Ki67 expression, a nuclear marker of proliferation (**Figure 1F**).

Metastatic lesions within the lungs, a common site of metastasis in metM-Wnt^lung^ bearing-mice, developed in 37.5% of the DIO control group and 12.5% in each of the other 2 groups (p = ns).

### 3.2 Advanced age and DIO each promote enrichment of inflammatory- and immune-related transcripts within tumor-adjacent adipose tissue

**Figure 2:**
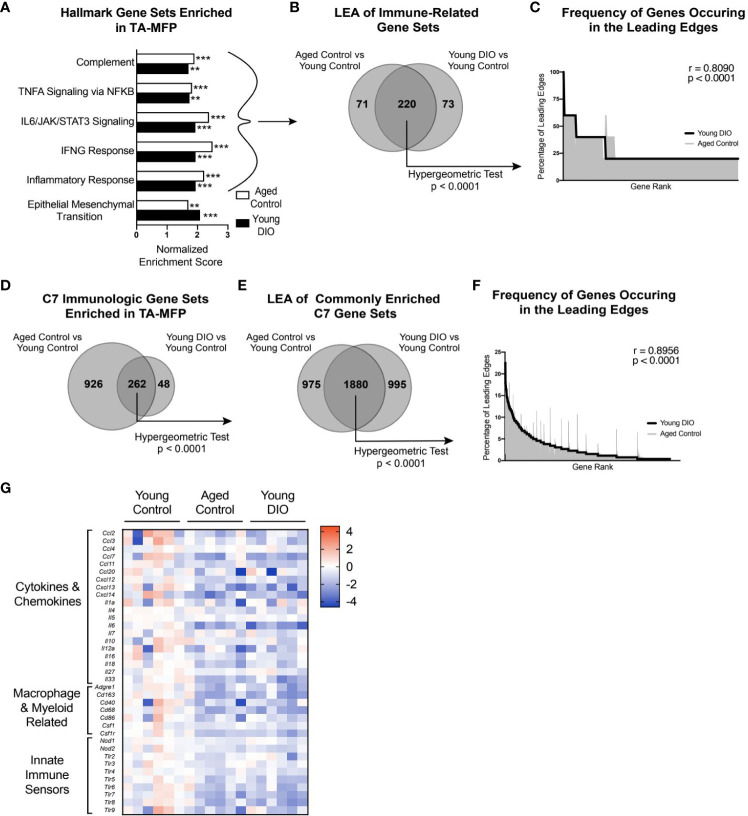
Advanced age and DIO induce convergent inflammatory alterations within the tumor-adjacent mammary adipose in a metM-Wnt^lung^ cell transplant model of TNBC. **(A)** Hallmark gene sets commonly enriched in tumor-adjacent mammary fat pad (TA-MFP) of aged control versus young control and young DIO vs young control mice determined following gene set enrichment analysis (GSEA). **(B)** Leading edge analysis (LEA) of immune-related Hallmark gene sets commonly enriched. **(C)** Frequency of genes occurring in the leading edge of commonly enriched, immune-related Hallmark gene sets versus gene rank. **(D)** C7 Immunologic gene sets commonly enriched in TA-MFP of aged control vs young control and young DIO vs young control micedetermined following GSEA. **(E)** LEA of all C7 gene sets commonly enriched. **(F)** Frequency of genes occurring in the leading edge of commonly enriched C7 gene sets versus gene rank. **(G)** Heat map of select leading edge genes. Significantly enriched gene sets defined as FDR q-value <0.05. Significance in overlap of leading edge genes analyzed using hypergeometric test. Correlation between the gene rank of leading edge contributors calculated by the Spearman test. Asterisks denote significance: **< 0.01, ***< 0.001.

To identify major cellular pathways and processes altered in the aged and obese TA-MFP, we performed GSEA with Hallmark gene sets. Compared with tumors from young control mice, tumors from aged control and young DIO mice were commonly enriched for gene sets related to immune signaling (e.g., TNFA Signaling *via* NFKB, IL6/JAK/STAT3 Signaling, and IFNG Response) and Epithelial Mesenchymal Transition, a process implicated in metastatic progression (**Figure 2A**). To determine genes that positively contributed to enrichment of immune-related gene sets in aged control and young DIO mice, relative to young control, LEA was performed. We observed significant overlap between the two leading edges, indicating that advanced age and DIO induce common immune-related transcriptional alterations within the TA-MFP (**Figure 2B**). Spearman correlation analysis of gene-level contributions to DIO-related leading edges versus age-related leading edges revealed a significant and strong correlation (**Figure 2C**).

Given the preponderance of immune-related gene sets enriched in the TA-MFP of both aged control and young DIO groups, relative to young control, we sought to determine whether this relationship was robust to further analysis using immunologic gene sets (C7 immunologic gene sets) (23).TA-MFP from aged control and young DIO mice were commonly enriched for 262 gene sets (**Figure 2D**). LEA identified significant overlap in age- and DIO-related leading edge genes (**Figure 2E**). Spearman correlation analysis revealed significantly concordant gene-level contributions to age- and DIO-related leading edges (**Figure 2F**). Overlapping leading edge genes (**Figure 2G**) included: 1) multiple chemokine, cytokine, and interleukin genes whose products would promote immune cell recruitment and stimulation within the mammary adipose; 2) genes related to macrophages and monocytes; and 3) numerous Toll-like and NOD-like receptor genes that can be activated by free fatty acids and damage-associated molecular proteins (10).

We also noted underrepresentation of gene sets in TA-MFPs from aged control and young DIO mice, compared with young control mice. Commonly underrepresented gene sets were related to adipogenesis and metabolism (e.g., Oxidative Phosphorylation and Fatty Acid Metabolism) suggesting differential regulation of these processes with advanced age and obesity (**Supplementary Figure 1A**). LEA revealed significant overlap in DIO- and age-related leading edges (**Supplementary Figure 1B**). Spearman correlation analysis of gene-level contributions to DIO-related leading edges versus age-related leading edges revealed a significant and strong correlation (**Supplementary Figure 1C**).

### 3.3 Advanced age and DIO each induce transcriptional changes within the tumor related to cellular proliferation, epithelial mesenchymal transition, and angiogenesis

Tumors from aged control and young DIO mice, relative to young control mice, were enriched for gene sets that reflect processes linked to tumor progression including cellular proliferation (e.g., mitotic spindle and E2F targets), epithelial mesenchymal transition, and angiogenesis, among others (**Supplementary Figure 1D**). LEA showed significant overlap in DIO- and age-related leading edge genes (**Supplementary Figure 1E**). Spearman correlation analysis of gene-level contributions to DIO-related leading edges versus age-related leading edges revealed a significant and strong correlation (**Supplementary Figure 1F**).

### 3.4 Advanced age and DIO each induce immunosuppression and reduce CD8+ T cell abundance within the tumor microenvironment

**Figure 3:**
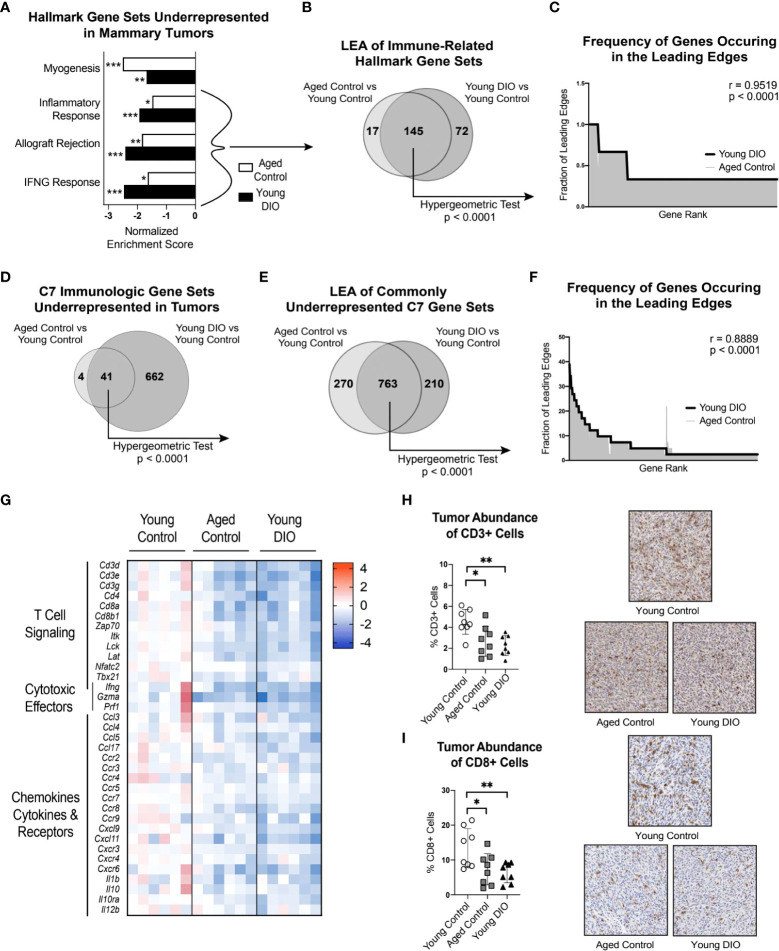
Advanced age and DIO promote immunosuppression within the tumor microenvironment and decreased CD8 T cell surveillance. **(A)** Hallmark gene sets commonly underrepresented in mammary tumors of aged control versus young control and young DIO versus young control mice determined following gene set enrichment analysis (GSEA). **(B)** Leading edge analysis (LEA) of immune-related Hallmark gene sets commonly underrepresented. **(C)** Frequency of genes occurring in the leading edge of commonly underrepresented, immune-related Hallmark gene sets versus gene rank. **(D)** C7 Immunologic gene sets commonly underrepresented in mammary tumor of aged control versus young control and young DIO versus young control mice determined following GSEA. **(E)** LEA of all C7 gene sets commonly underrepresented. **(F)** Frequency of genes occurring in the leading edge of underrepresented C7 gene sets versus gene rank. **(G)** Heat map of select leading edge genes. **(H)** Percentage of cells stained positive for CD3 and **(I)** CD8 analyzed using QuPath and presented as mean ± SD. Significantly enriched gene sets defined as FDR q-value <0.05. Significance in overlap of leading edge genes analyzed using hypergeometric test. Correlation between the gene rank of leading edge contributors calculated by the Spearman test. Immunohistochemical staining analyzed relative to young control using one-way ANOVA and Dunnet *post hoc* test. Asterisks denote significance: *< 0.05, **< 0.01, ***< 0.001.

Contrasting the TA-MFP, mammary tumors from aged control and young DIO mice were underrepresented for immune-related gene sets(i.e., Inflammatory Response, Allograft Rejection, and IFNG Response; **Figure 3A**), relative to young control mice. LEA of immune-related gene sets revealed significant overlap in leading edge genes (**Figure 3B**). Spearman correlation analysis of gene-level contributions to DIO-related leading edges versus age-related leading edges revealed a significant and strong correlation (**Figure 3C**). GSEA with C7 immunologic gene sets revealed that tumors from aged control and young DIO mice, relative to young control mice, were commonly underrepresented for 41 genes sets (**Figure 3D**). LEA showed significant overlap in leading edge genes (**Figure 3E**). Spearman correlation analysis of gene-level contributions to DIO-related leading edges versus age-related leading edges revealed a significant and strong correlation (**Figure 3F**).

Many of the leading edge tumoral genes underrepresented in aged control and young DIO mice, relative to young control mice, related to T cell receptor signaling, immune, and cytotoxic effector function (**Figure 3G**). We were particularly intrigued by underrepresentation of genes encoding CD3 and CD8 receptors as they are markers of cytotoxic CD8+ T cells. Therefore, we used IHC analysis to test whether differential gene expression translated to differences in CD3+ and CD8+ cell abundance within mammary tumors. Mammary tumors from aged control and young DIO mice, relative to young control mice, showed significantly lower percentages of CD3+ and CD8+ cells (**Figures 3H, I**).

### 3.5 Reduction of CD8+ T cell immune surveillance enhances growth of metM-Wnt^lung^ tumor cells *in vivo*

**Figure 4:**
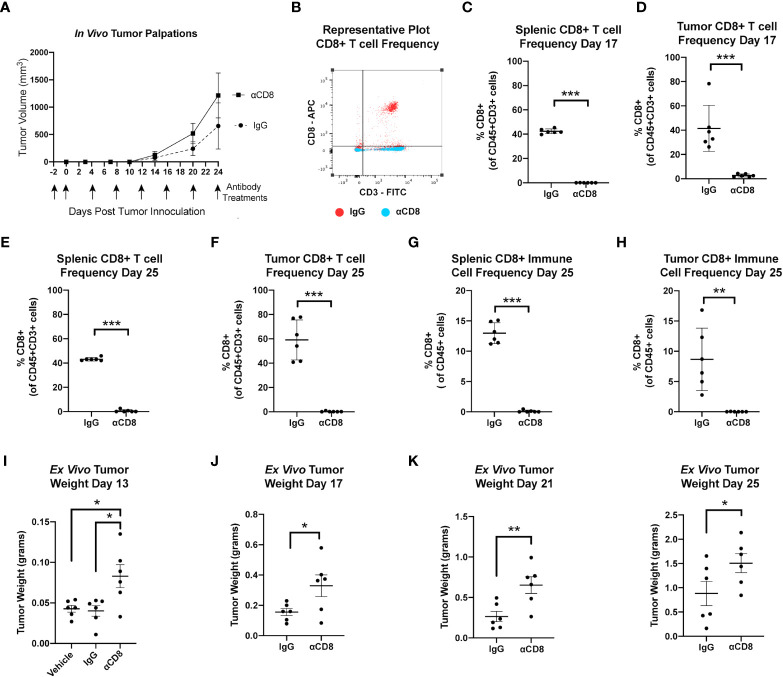
MetM-WNT^lung^ tumor growth is constrained by CD8 T cell immune surveillance. **(A)** Study schematic and *in vivo* tumor volume. **(B)** Representative flow cytometry plot quantifying CD8+ T cells from IgG treated (red) and anti-CD8 antibody treated (blue) mice. **(C-F)** Flow cytometric analysis of splenic and tumoral CD45+CD3+CD8+ T cell populations at interim timepoint and study end point. **(G, H)** Flow cytometric analysis of splenic and tumoral CD45+CD8+ cells at study endpoint. **(I-K)** *Ex vivo* tumor mass measured days 13, 17, 21, and 25 following tumor injections. Data presented as mean ± SD. Differences across three groups analyzed using one-way ANOVA and Tukey *post hoc* test. Statistical differences across two groups determined by t test. Asterisks denote significance: *p< 0.05, **p< 0.01, ***p< 0.001.

These findings suggest that obesity and advanced age enhance mammary tumor progression through reduced CD8 T cell-mediated immune surveillance within the mammary tumor microenvironment. To test if CD8 T cell surveillance constrains growth in the metM-Wnt^lung^ cell-driven model of TNBC, female C57BL/6J mice were treated with either anti-mouse CD8α-depleting antibody, rat IgG2B isotope control, or a vehicle control beginning two days prior to mammary injection with MetM-WnT^lung^ cells (**Figure 4A**). Flow cytometric analysis at an interim timepoint (17 days after tumor inoculation) and study endpoint (25 days after tumor inoculation) confirmed depletion of CD3+CD8+ T cells in the spleens and tumors of CD8α-treated mice compared to IgG2B isotope-treated control (**Figures 4B-E**). As anticipated, negligible CD8 was detected within the total CD45+ immune cell populations isolated from spleens and tumors of αCD8-treated mice (**Figures 4F, G**). Depletion of CD8 T cells augmented metM-WnT^lung^ cell growth, resulting in greater *ex vivo* tumor mass among CD8α-treated mice at all measured timepoints (13, 17, 21 and 25 days following tumor injection) compared to isotope-treated controls (**Figures 4H-K**).

### 3.6 Combination of advanced age and DIO exacerbates mammary tumor growth in a E0771 cell transplant model of TNBC

**Figure 5:**
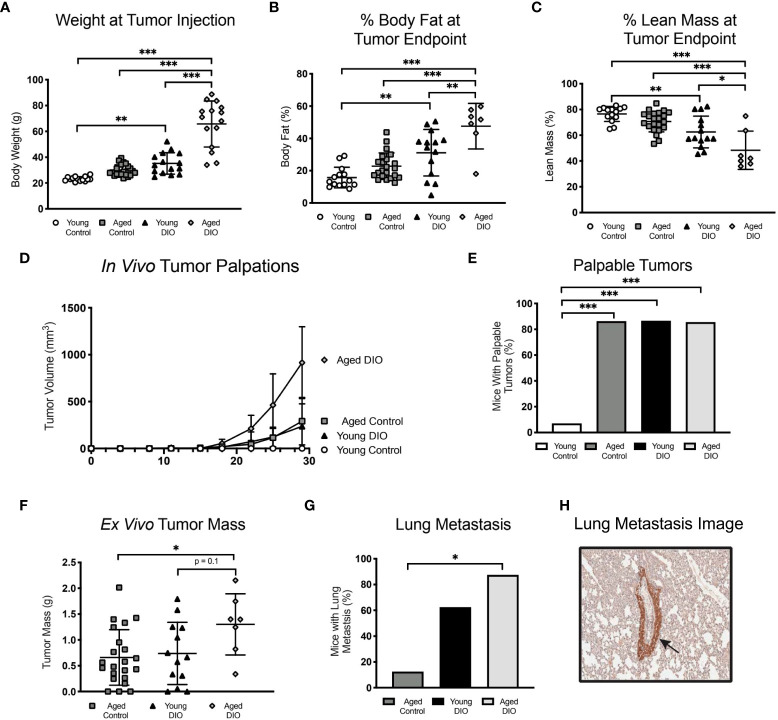
Advanced age and DIO exert a combined effect on mammary tumor progression in a E0771 cell transplant model of TNBC. **(A)** Body weight at tumor inoculation. **(B)** % body fat mass and **(C)** % lean mass measured at study endpoint. **(D)** *In vivo* tumor volume following E0771 cell injection. **(E)** Percentage of mice that developed palpable mammary tumors, analyzed using Fisher’s exact test. **(F)** Ex vivo tumor mass. Only one mouse in the young control group developed a tumor, so no data is shown for that group. **(G)** Percentage of mice that developed at least one metastatic lesion in the lung, statistical difference analyzed using Fisher’s exact test. Only one mouse in the young control group developed a primary tumor with no metastatic lesions,so no data is shown for that group. **(H)** R epresentative image of GFP stained metastatic lesion (arrow). Data presented as mean ± SD. Differences across groups analyzed using one-way ANOVA and Tukey *post hoc* test, unless otherwise specified. Asterisks indicate differences in significance. *p < 0.05, **p < 0.01, ***p < 0.001.

Having demonstrated that advanced age and obesity separately promote tumor growth in a metM-WnT^lung^ cell-driven model of TNBC, we sought to confirm these findings in a different, E0771 cell transplant model and to test if the combination of advanced age and obesity further exacerbates tumor growth. In a 2x2 factorial study design, groups of young control, young DIO, aged control and aged DIO mice were engrafted with E0771 tumor cells and monitored for a month for tumor progression. Advanced age alone did not alter body weight or body composition measures in aged control mice compared to young control (**Figures 5A-C**). In contrast, DIO feeding resulted in significantly greater body weightand percent body fat mass relative to aged-matched control fed mice (**Figures 5A, B** **)**. Further, the combination of advanced age and DIO feeding resulted in the greatest body weight and percent body fat mass, relative to all other groups (**Figures 5A, B** **)**. DIO feeding increased total lean mass relative to aged-matched control mice; however, changes in adiposity were greater resulting in an overall reduction in percent lean mass (**Figure 5C**).

Advanced age and obesity—alone and in combination—increased tumor burden, as aged control, young DIO, and aged DIO mice all exhibited increased tumor engraftment relative to young control mice (**Figures 5D, E** **)**. Only one young control mouse developed a palpable tumor. This mouse also developed severe dermatitis and had to be euthanized prior to tumor endpoint. Due to their minimal tumor progression, young control mice were excluded from subsequent analyses. Aged DIO mice had significantly greater *ex vivo* tumor mass and metastatic burden than aged control mice (**Figures 5F, G** **)**. Differences in tumor mass and metastases between aged DIO and young DIO did not reach statistical significance (**Figures 5F–H** **)**.

### 3.7 Obesity combined with advanced age heightens inflammatory perturbations and tumor immune suppression more than advanced age alone in an E0771 cell transplant model of TNBC

**Figure 6:**
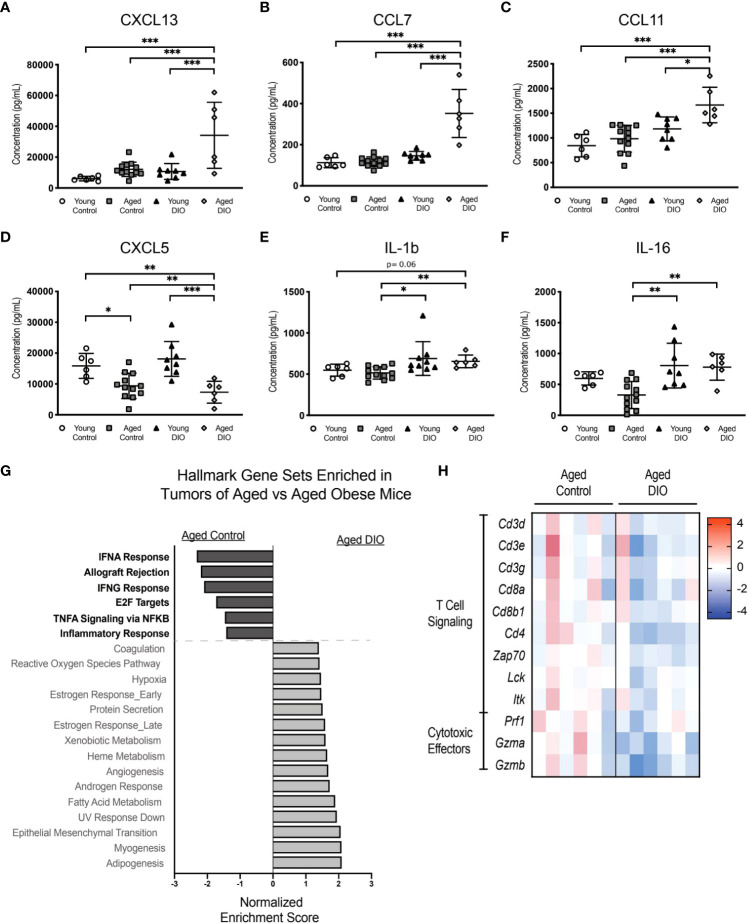
The combination of advanced age and DIO exacerbates tumor immune suppression. **(A-F)** Serum cytokines measured in serum collected prior to tumor inoculation. **(G)** Hallmark gene sets commonly enriched and underrepresented in mammary tumor of aged DIO versus aged control mice determined using gene set enrichment analysis (GSEA). **(H)** Heat map of select leading edge genes. Data presented as mean ± SD. Differences across groups analyzed using one-way ANOVA and Tukey *post hoc* test, unless otherwise specified. Significantly enriched gene sets defined as FDR q-value <0.05. Asterisks indicate differences in significance. *p < 0.05, **p < 0.01, ***p < 0.001.

Given that advanced age combined with obesity increased transplanted E0771 tumor burden in aged DIO mice to a greater extent than either advanced age or DIO alone, we assessed whether levels of tumor-promoting serum cytokines and/or systemic inflammation were increased in aged DIO mice, relative to all other groups. Levels of 32 cytokines were measured in serum collected prior to tumor injection. The levels of six cytokines differed among groups (**Figures 6A-F** **),** including significant elevations in aged DIO mice, relative to the other groups, in the tumor-promoting chemokines CXCL13, CCL7, and CCL11 (**Figures 6A-C**) (24–26).

Since obesity combined with advanced age accelerated primary tumor growth and metastatic burden more so than advanced age alone, we postulated that aged DIO mice would have a more immunosuppressive tumor microenvironment than aged control mice. Tumor transcriptional data was collected, and pairwise pathway enrichment between these two groups was determined using GSEA and Hallmark gene sets. Immune-related Hallmark gene sets were underrepresented in tumors from aged DIO mice, relative to aged control mice (**Figure 6G**). Underrepresented gene sets included Allograft Rejection, IFNG response, and Inflammatory Response, gene sets that were also underrepresented in tumors from aged control and young DIO mice, compared to young control mice, in Study A (**Figure 3A** **)**. LEA revealed that tumors from aged DIO mice, relative to aged controls, were underrepresented for genes relating to T cell receptor signaling and cytotoxic effector function (**Figure 6H**).

## 4 Discussion

Our findings are the first to demonstrate that advanced age accelerates tumor progression in 2 independent preclinical models of TNBC. We have previously shown that DIO exerts procancer effects across a variety of cancer types (7, 8, 13) and that mammary adipose inflammation and tumor immunosuppression are major contributors to DIO’s effects in BC. We now further report that the combination of advanced age and obesity exerts greater procancer effects than either advanced age or obesity alone in a preclinical model of TNBC. We also newly report an overlap in advanced age- and obesity-related biology in tissues that have previously been implicated in obesity-associated BC progression: the mammary tumor and tumor-adjacent mammary adipose. Finally, *via* a mechanistic study, we demonstrate that differential abundance of CD8+ T cells could explain differential tumor progression in our models.

At study endpoint, aged control (18 months old) and young DIO (8 months old) mice exhibited increased intramammary engraftment of basal-like E0771 cells and enhanced growth of claudin-low metM-Wnt^lung^ cells when compared with young control (8 months old) mice. This confirms previous reports from our lab as well as others demonstrating tumor-promoting effects of DIO in mouse models of BC (7, 8, 13). On the other hand, relatively few studies have investigated the effects of age on mammary tumor growth. One prior study tested whether multiple mammary carcinoma cells exhibited differential growth kinetics in older (12 months old) versus younger (8-10 weeks old) mice (27). At early timepoints, 4T1 tumors were significantly larger in older mice; however, at endpoint there was no significant difference in tumor size in the aged compared with younger mice (27). In contrast, Met1 tumors grew more slowly in older mice and produced significantly smaller tumors, compared with younger mice at 30 days post tumor inoculation (27). Investigation in three additional cell lines confirmed strain-specific differences with two cell lines (4T07 and 67NR) having similar growth, and one (McNeuA) having slower growth, in older compared with younger mice (27).

The differential impact of advanced age in our study, compared with observations in the aforementioned study (27), suggests varying age-related effects across tumor cell lines and BC subtypes. There could also be biological differences in 17-month-old, compared with 12-month-old, mice that promote tumorigenesis. For example, cellular senescence, a marker of chronological age, increases with advancing age and as a result would be elevated in 17-month-old compared 12-month-old mice (28, 29). Senescent cells often express a senescence-associated secretory phenotype (SASP), composed of inflammatory factors (e.g., IL-6 and IL-1β), which also increase with age and contribute to age-related inflammation (known as inflammaging) (30). Furthermore, cellular senescence and SASP mechanistically contribute to progression of BC by promoting the growth of preneoplastic and neoplastic cells, increasing metastasis, and decreasing chemotherapeutic response (31, 32). Given that BC is more prevalent in women of advanced age and the differential impact of advanced age-related biology on tumor growth in our models of TNBC, it will be important for future preclinical studies to consider age in their study design and data interpretation.

In our study, acceleration of tumor growth with advanced age and obesity – alone and in combination - can be explained, in part, by convergent inflammatory- and immune-related transcriptional patterns in the TA-MFP. Notably, each advanced age and obesity resulted in enrichment of IL-6/JAK/STAT3 signaling, which cultivates an immunosuppressive environment by stimulating expansion of myeloid-derived suppressor cells and controlling antitumor T cell responses (33, 34). Similarly, elevated IL-6 is observed in adipose tissue of aged and obese women and is associated with poor prognosis in BC patients (14, 35–38). Thus, advanced age and obesity each promote immunosuppressive signaling within the mammary adipose adjacent to mammary tumor cells, which could contribute to accelerated tumor progression through paracrine exchange of immunosuppressive molecules and suppression of cells that participate in the antitumor immune response.

We found that inflammatory and immunosuppressive signaling within the TA-MFP was associated with tumor immunosuppression in aged control and young DIO mice. Specifically, aged control and young DIO tumors, relative to young control tumors, were underrepresented for inflammation- and immune-related gene sets including IFNG response. Similarly, DIO in the context of advanced age further exacerbated immunosuppression as tumors from aged DIO mice, relative to aged control mice, were underrepresented for inflammation- and immune-related gene sets, again including IFNG response. IFN-γ is a critical antitumor immunity mediator that is secreted by a variety of cell types, including CD8 T cells, and results in the upregulation of antigen presentation machinery and promotion of cytotoxic CD8 T cell differentiation and cytotoxic effector function (39). In our study, LEA of commonly underrepresented immune-related gene sets in tumors of aged control and young DIO mice, compared with young control mice, as well as aged DIO mice, compared with aged control mice, revealed that suppressed transcripts were related to cytotoxic CD8 T cell signaling and cytotoxic effector functions. Aligning with transcriptional changes, IHC analysis demonstrated reduced abundance of CD8 cells within the tumor microenvironment. Taken together, these findings indicate that advanced age and obesity, alone and in combination, reduce CD8 T cell abundance within mammary tumors and suppress transcripts relating to antitumor immunity.

In patients with BC, decreased abundance of tumor-infiltrating CD8 T cells are associated with diminished overall survival and relapse free survival (40). Preclinical studies have mechanistically proven that CD8 T cells control tumor growth across many cancer models, including the E0771 transplant TNBC model utilized in this study (41). We tested if CD8 T cells impacted growth in the metM-WnT^lung^ cell-driven TNBC model and found a similar CD8 T cell-mediated constraint of tumor growth. Thus, it is conceivable that reduced CD8 T cell surveillance within the tumors of aged control and young DIO mice, relative to young control mice, significantly contributed to observed acceleration in mammary tumor growth.

Limitations of this study include the use of murine models to study human BC as biological differences exists between the two species. However, literature supports conservation of certain aspects of age- and obesity-associated immune dysfunction across species (12, 23). Nevertheless, more translational research efforts are needed to determine whether these preclinical findings are consistent with the effects of human aging and obesity. In addition, due to low engraftment in young control mice in the E0771 transplant model, we were unable to fully analyze the interactive effects of advanced age and obesity on TNBC. Given the average lifespan of female C57BL/6 is ~27 months, we also experienced reduced numbers of aged DIO mice, relative to the other groups, completing the study to tumor endpoint due to various adverse health effects associated with the combination of obesity and advanced aging near the upper range of this strain’s lifespan. In conclusion, these findings reveal striking similarities in advanced age- and obesity-related biology that contributes to accelerated mammary tumor progression. The differential effects of advanced age and obesity on the mammary tumor microenvironment may necessitate alternative therapeutic approaches for patients with TNBC and advanced age, obesity, or the combination of advanced age and obesity. Further research is needed to fully elucidate mechanisms underlying advanced age- and obesity-related BC progression and aid in the development of mechanism-based strategies for preventing or controlling BC.

## Data availability statement

The original contributions presented in the study are included in the article/**Supplementary Material**. Further inquiries can be directed to the corresponding author.

## Ethics statement

The animal study was reviewed and approved by University of North Carolina at Chapel Hill IACUC Committee.

## Author contributions

LS, MR, LB, SBM and SH contributed to conception and design of the study. LS, DC, MR, AC, MSC, EG, NS, SBM, ER and MFC contributed to the generation of data. LS, LB, EG and MC performed the statistical analysis. SM performed histopathologic analysis. LB and SH wrote the first draft of the manuscript. All authors contributed to the article and approved the submitted version. 

## Supplementary material

Supplementary Figure 1 Advanced age and DIO induce overlapping TA-MFP transcriptional alterations related to regulation of metabolic processes and enrich the tumor transcriptome for signaling pathways supportive of tumor growth. **(A)** Hallmark gene sets commonly underrepresented in the tumor adjacent mammary fat pad (TA-MFP) of aged control versus young control mice and young DIO versus young control mice determined following gene set enrichment analysis (GSEA). **(B)** Leading edge analysis (LEA) of Hallmark gene sets commonly underrepresented in TA-MFP. **(C)** Frequency of the genes occurring in the leading edge of Hallmark gene sets commonly underrepresented in the TA-MFP versus gene rank. **(D)** Hallmark gene sets commonly enriched in mammary tumor of aged control versus young control and young DIO versus young control mice determined following gene set enrichment analysis (GSEA). **(E)** Leading edge analysis (LEA) of Hallmark gene sets commonly enriched in mammary tumors. **(F)** Frequency of genes occurring in the leading edge of Hallmark gene sets commonly enriched in mammary tumors verses gene rank. Significant gene set enrichment defined as FDR q-val <0.05. Asterisks denote significance: *< 0.05, **< 0.01, ***< 0.001. Significance in overlap of leading edge genes analyzed using hypergeometric test. Correlation between the gene rank of leading edge contributors calculated by the Spearman test.
