# Supplementary figures and images for: Separate and combined effects of advanced age and obesity on mammary adipose inflammation, immunosuppression and tumor progression in mouse models of triple negative breast cancer
Source: Front Oncol. 2023 Jan 4;12:1031174. doi: 10.3389/fonc.2022.1031174 (PMC9846347; doi:10.3389/fonc.2022.1031174)

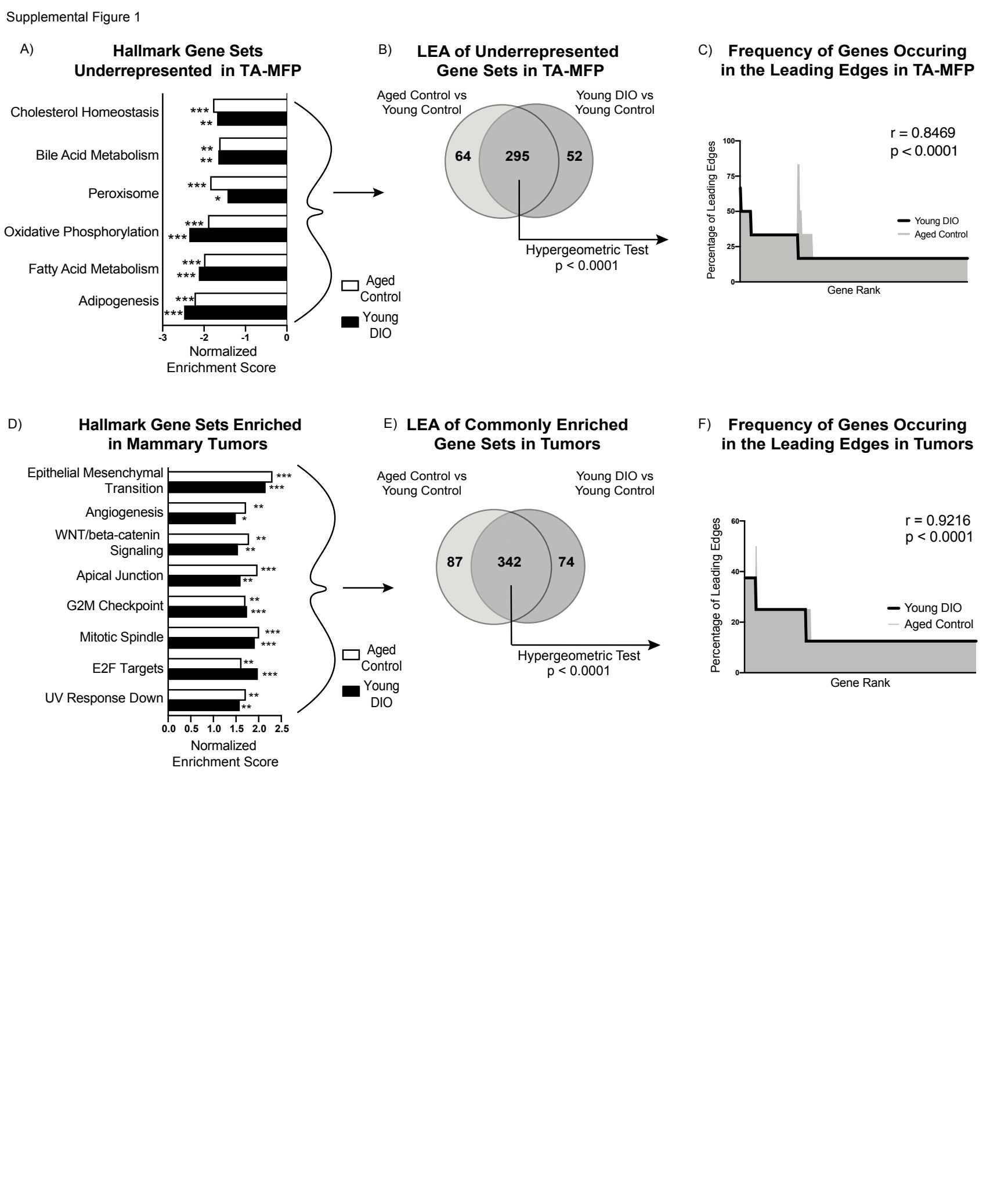

Supplement: Supplementary Figure 1 — Advanced age and DIO induce overlapping TA-MFP transcriptional alterations related to regulation of metabolic processes and enrich the tumor transcriptome for signaling pathways supportive of tumor growth. (A) Hallmark gene sets commonly underrepresented in the tumor adjacent mammary fat pad (TA-MFP) of aged control versus young control mice and young DIO versus young control mice determined following gene set enrichment analysis (GSEA). (B) Leading edge analysis (LEA) of Hallmark gene sets commonly underrepresented in TA-MFP. (C) Frequency of the genes occurring in the leading edge of Hallmark gene sets commonly underrepresented in the TA-MFP versus gene rank. (D) Hallmark gene sets commonly enriched in mammary tumor of aged control versus young control and young DIO versus young control mice determined following gene set enrichment analysis (GSEA). (E) Leading edge analysis (LEA) of Hallmark gene sets commonly enriched in mammary tumors. (F) Frequency of genes occurring in the leading edge of Hallmark gene sets commonly enriched in mammary tumors verses gene rank. Significant gene set enrichment defined as FDR q-val <0.05. Asterisks denote significance: *< 0.05, **< 0.01, ***< 0.001. Significance in overlap of leading edge genes analyzed using hypergeometric test. Correlation between the gene rank of leading edge contributors calculated by the Spearman test. [file Image_1.jpeg]
